# Neural Circuitry for Stress Information of Environmental and Internal Odor Worlds

**DOI:** 10.3389/fnbeh.2022.943647

**Published:** 2022-06-16

**Authors:** Kensaku Mori, Hitoshi Sakano

**Affiliations:** ^1^RIKEN Center for Brain Science, Wako, Japan; ^2^Department of Brain Function, School of Medical Sciences, University of Fukui, Fukui, Japan

**Keywords:** olfactory perception, duplicated glomerular maps, exteroceptive and interoceptive odors, inhalation and exhalation, innate vs. learned decisions, olfactory stress signals, neural circuits

## Abstract

In mammals, odor information detected in the olfactory epithelium is converted to a topographic map of activated glomeruli in the olfactory bulb. Odor signals are then conveyed by projection neurons to the olfactory cortex for decision making. Odor information is processed by two distinct pathways, one is innate and the other is learned, which are separately activated during exhalation and inhalation, respectively. There are two types of odor signals, exteroceptive and interoceptive, which are also processed in different phases of respiration. Exteroceptive sensory information whether attractive/pleasant or aversive/stressful, is evaluated by the valence regions in the amygdala. Stress is an alert signal telling the body to take an action so that the normal condition can be recovered. When the odor quality is negative, the brain sets up a behavioral strategy to avoid the danger or to improve the situation. In this review article, we will describe the recent progress in the study of olfactory perception focusing on stress responses to external and internal odors.

## Introduction

**FIGURE 1:**
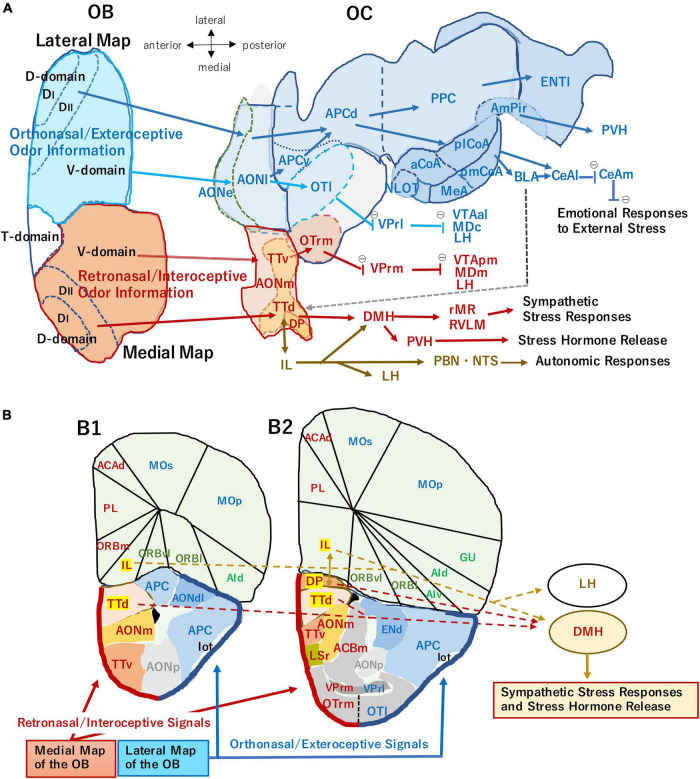
**(A)** An unfolded map (ventral center view) of the mouse olfactory bulb (OB) and olfactory cortex (OC) illustrating two olfactory processing streams. The medial stream (magenta arrows) originates from the medial map of the OB (dark orange) and conveys retronasal/interoceptive information to the medial part of the anterior olfactory nucleus (AONm), ventral tenia tecta (TTv), dorsal tenia tecta (TTd), dorsal peduncular cortex (DP), and rostromedial part of olfactory tubercle (OTrm). OTrm sends inhibitory axons (marked by θ) to the rostromedial part of ventral pallidum (VPrm). The VPrm projects inhibitory axons to the posteromedial part of ventral tegmental area (VTApm), medial part of mediodorsal nucleus in the thalamus (MDm), and lateral hypothalamus (LH). The TTd and DP project to the dorsomedial nucleus of hypothalamus (DMH), which projects to the sympathetic premotor neurons in the rostral medullary raphe region (rMR) and rostral ventrolateral medulla (RVLM) to generate sympathetic responses. The TTd and DP have reciprocal connections with infralimbic cortex (IL), which projects to the autonomic centers in the hypothalamus and brainstem, such as DMH, LH, parabrachial nucleus (PBN), and nucleus of solitary tract (NTS). The ventral part of DMH projects to the paraventricular nucleus of hypothalamus (PVH) and activates the hypothalamo-pituitary-adrenal axis to release stress hormones (Herman et al., 2016; Nakamura and Morrison, 2022). The lateral stream (blue arrows) originates from the lateral map of the OB (blue) and sends orthonasal/exteroceptive odor information to the external and lateral parts of the anterior olfactory nucleus (AONe, AONl), lateral part of the olfactory tubercle (OTl), ventral and dorsal parts of the anterior piriform cortex (APCv, APCd), posterior piriform cortex (PPC), nucleus of the lateral olfactory tract (NLOT), anterior, posterolateral, and posteromedial nuclei of the cortical amygdala (aCoA, plCoA, pmCoA), medial amygdala (MeA), amygdalo-piriform transition area (AmPir), and lateral entorhinal area (ENTl). The CoA projects to the basolateral amygdaloid complex (BLA) and lateral and medial nuclei of central amygdala (CeAl, CeAm) to induce emotional responses. A gray broken arrow from the amygdala to the DP/TTd indicates a putative route for the transmission of aversive orthonasal odor information to the DP/TTd-DMH pathway. The OTrl sends inhibitory axons to the rostrolateral part of ventral pallidum (VPrl). The VPrl projects inhibitory axons to the anterolateral part of ventral tegmental area (VTAal), the central part of mediodorsal nucleus in the thalamus (MDc), and LH. The unfolded map was modified from Mori and Manabe (2014), with permission from Springer Nature. **(B)** Medial and lateral areas of the OC shown on the coronal section through the olfactory peduncle (B1) and that through the rostral part of the OT (B2). Thick magenta line indicates the superficial layer of the medial OC mainly receiving retronasal information from the medial OB map. Thick blue line indicates the superficial layer of the lateral OC mainly receiving orthonasal information from the lateral OB map. The IL, DP, and TTd (highlighted) form the ventromedial prefrontal cortex, and their projection to the hypothalamus is shown by broken arrows. ACAd, dorsal part of anterior cingulate area; ACBm, medial part of accumbens nucleus; AId, dorsal part of agranular insular cortex; AIv, ventral part of agranular insular cortex; AONdl, dorsolateral part of anterior olfactory nucleus; AONp, posterior part of anterior olfactory nucleus; ENd, dorsal endopiriform nucleus; Gu, gustatory cortex; lot, lateral olfactory tract; LSr, rostral part of lateral septum; MOp, primary motor cortex; MOs, secondary motor cortex; ORBl, lateral part of orbital cortex; ORBm, medial part of orbital cortex; ORBvl, ventrolateral part of orbital cortex; OTl, lateral part of olfactory tubercle; OTrm, rostromedial part of olfactory tubercle; PL, prelimbic area; VPrl, rostrolateral part of ventral pallidum; VPrm, rostromedal part of ventral pallidum. The contour of each area is generated using Allen Brain Atlas (https://atlas.brain-map.org), the Allen Institute for Brain Science (2004).

In mice, odor signals detected by olfactory receptors (ORs) are converted to an odor map, a combinatorial pattern of activated glomeruli (Mori and Sakano, 2011). The glomerular map in the olfactory bulb (OB) is generated by distinct sets of axon guidance/sorting molecules expressed in olfactory sensory neurons (OSNs) (Sakano, 2010). The OB is not merely a projection screen for odor maps, but is also composed of functional domains for different odor qualities. Odor information detected in the olfactory epithelium (OE) is roughly sorted into two separate qualities, stressful (aversive) and pleasant (attractive) during the process of primary projection (Inokuchi et al., 2017). Aversive information is represented in the dorsal domain of the OB (Figure 1A) and transmitted to the negative valence region in the amygdala (Miyamichi et al., 2011; Root et al., 2014). In contrast, attractive information is collected in the postero-ventral domain and transmitted to the positive valence regions in the amygdala and olfactory tubercle (OT) (Inokuchi et al., 2017; Midroit et al., 2021) to elicit emotional and behavioral responses.

Odor information comes from not only the outside environment but also from inside the body. Exteroceptive/orthonasal odors are essential in searching food, finding for mating-partners, and detecting dangers. Interoceptive/retronasal odors are important for evaluating the quality of chewing food, whether it should be swallowed or not. Furthermore, retronasal odor is useful for checking the physiological condition of the body. In the OB, there are two sets of mirror-symmetrical sensory maps, lateral and medial, that may be separately used for interpreting outside and inside odor information, respectively (Figure 1; Mori and Sakano, 2022).

Processing of odor signals is closely related to the respiratory cycle: inhalation phase is for exteroceptive inputs, while exhalation phase is for interoceptive inputs. Olfactory information is processed *via* two distinct neural pathways: the direct pathway for innate decisions and the multi-synaptic pathway for memory-based learned decisions (Mori and Sakano, 2022). These circuits are differentially activated during respiration by separate subsets of projection neurons, mitral cells (MCs) and tufted cells (TCs). For odor identification and recollection of associated memory, map information is transmitted from the OB to the anterior olfactory nucleus (AON) by TCs. In contrast, for innate decisions, odor signals are directly delivered to the amygdala by MCs.

Emotional status is generated by various hormones and neuro-transmitters based on the sensory quality. When it is comfortable, the mind becomes pleasant and relaxed, but when it is not, the body feels stress. Stress is a signal alerting the animal that the environmental situation or inner-body condition is deviated from its normal status, and that it needs to take an action to avoid the danger or improve the situation. To cope with distinct environmental and internal challenges, the brain chooses appropriate behavioral responses and coordinates them with neuroendocrine and autonomic responses (Ulrich-Lai and Herman, 2009). In this review article, we will summarize the recent progress in the study of olfactory perception focusing on the neural circuitry for external and internal odor information.

## Olfactory Map Formation in the Olfactory Bulb

In rodents, there are ∼1000 OR species individually expressed in ∼300,000 OSNs (Buck and Axel, 1991). Each OSN expresses only one functional OR gene in a mono-allelic manner. This one neuron/one receptor rule is ensured by stochastic activation of OR genes with *cis*-acting enhancers and by negative feedback regulation with functional OR molecules (Serizawa et al., 2003). Furthermore, OSNs expressing the same OR species converge to a specific target site forming a glomerular structure (Mombaerts et al., 1996), which is referred to as the one glomerulus/one receptor rule. Thus, an olfactory map contains ∼1000 glomeruli individually representing one OR species. In each OB, right and left, there are two mirror-symmetrical olfactory maps (Figure 1). It has long been puzzling why two redundant maps are needed for the mammalian olfactory system.

In olfactory map formation, two sets of axon-targeting molecules are involved: one for the dorsal/ventral (D/V) axis and the other for the anterior/posterior (A/P) axis of the OB (Sakano, 2010). For D/V targeting, there is a close correlation between the locations of OSNs in the OE and their target sites in the OB. At least, two pairs of axon guidance molecules are known to be involved in D/V targeting: they are Neuropilin 2 (Nrp2)/Semaphorin 3F (Sema3F) and Robo2/Slit1 (Takeuchi et al., 2010). A characteristic feature of olfactory map formation is that OR molecules play an instructive role in guiding OSN axons to the target glomeruli. OR-derived cAMP, whose levels are uniquely determined by OR species, is responsible for regulating the transcription levels of A/P targeting molecules, e.g., Nrp1, Sem3A, and Plexin A1 (PlxnA1) (Imai et al., 2006). G-protein-coupled receptors (GPCRs) including ORs possess two different conformations, active and inactive, flipping spontaneously between the two states in the absence of ligands (Rasmussen et al., 2011). Agonist-independent GPCR activity generated by this conformational change is used to regulate A/P targeting molecules using cAMP as a second messenger (Nakashima et al., 2013).

In immature OSNs, a coarse map is generated according to a genetic program by a combination of D/V and A/P targeting. However, the map still needs to be refined in an activity-dependent manner. Both attractive (e.g., Kirrel2 and Kirrel3) and repulsive (e.g., ephrinA and EphA) axon-sorting molecules are involved, whose expression levels are determined by OR-specific neuronal activity with cAMP (Serizawa et al., 2006). How is it then that the two types of regulation, activity-dependent and independent, are separately controlled in OSNs using the same OR identity and cAMP? Activity-independent A/P targeting is regulated in immature OSNs by the base-line activity of ORs using the G protein, G_s_, whereas glomerular segregation is controlled in mature OSNs by firing spikes with a different type of G protein, G_olf_ (Nakashima et al., 2013; Nishizumi and Sakano, 2019).

## How Is the Olfactory Map Interpreted for Behavioral Decisions?

Olfactory information detected in the OE is converted to a combinatorial pattern of firing glomeruli and is also sorted into distinct odor qualities forming functional domains in the OB (Mori and Sakano, 2011). Odor signals are further transmitted to the olfactory cortex (OC) and amygdala for making behavioral decisions. There are two separate neural circuits to evaluate odor quality, innate and learned, using distinct types of projection neurons, MCs and TCs. Existence of the innate olfactory circuit was elegantly shown by Kobayakawa et al. (2007) using the domain-specific ablation mice. Deletion mapping for the functional domains revealed that the fear domain (D_II_) is located in the postero-dorsal OB whereas the aversive domain (D_I_) is separately mapped next to the D_II_ (Figure 1). In the dorsal-domain ablated mice (ΔD), fear responses to a fox odor, trimethyl thiazoline (TMT), are abolished. However, the ΔD mice can learn fear to TMT if TMT is presented with an injection of LiCl, which induces malaise. The ΔD mice are also capable of discriminating two structurally related odorants, e.g., pentanal and hexanal.

For identification of odors, an odor map is transmitted from the OB to the AON by a specific subset of TCs maintaining its topographic orders (Mori and Sakano, 2021). This odor map appears to be recognized as a QR code in the OC to discriminate odor species and to recollect the memory-scene in the previous odor experience. We assume that the recalled memory engram further activates the associated valence network, so that the odor quality is evaluated for learned decisions.

How about innate decisions for instinct odor responses? The fox odor TMT activates more than 20 glomeruli in the OB. Although many of them are located in the D_I_ and D_II_ domains, it was not clear whether the activation pattern of glomeruli in D_II_ induces fear, or if each individual glomeruli possess specific functions. In order to address this question, Saito et al. (2017) analyzed TMT-responsive ORs whose glomeruli are located in the dorsal OB. Among them, Olfr1019 was chosen because it demonstrated the highest ligand reactivity and selectivity, and its glomerulus was located not in the avoidance (D_I_) but in the fear (D_II_) domain. For optogenetic experiments, the channel rhodopsin gene was inserted into *Olfr1019*. Interestingly, photo-illumination of the knock-in (KI) mice induced immobility (freezing), but not avoidance behavior. Furthermore, the postero-medial part of the cortical amygdala (pmCoA) and amygdalo-piriform transition area (AmPir) were not activated in the photo-illuminated KI: the pmCoA and AmPir are responsible for inducing avoidance behavior (Root et al., 2014) and for releasing the stress hormone ACTH (Kondoh et al., 2016), respectively. In agreement with the KI experiments, the knock-out (KO) of *Olfr1019* did not affect stress responses with ACTH (Saito et al., 2017). These observations demonstrate that TMT-induced fear can be divided into two separate components, immobility and avoidance. In addition, TMT-induced freezing is not an ACTH-secreting stress response, but an active behavioral decision to not move. It will be interesting to study what kind of stress signals other than TMT are detected in the D_II_ domain. In any case, it appears that once the MCs in a particular OB domain are activated, even *via* a single glomerular structure, a specific innate response can be induced without regard to the odor specificity.

## Direct Pathways to the Amygdala for Innate Olfactory Decisions

Olfactory information is recognized as an odor map to identify the input odor for recollection of the associated scene. Separately from this multi-synaptic pathway, input signals are directly conveyed by MCs to the distinct valence regions in the amygdala for innate olfactory decisions (Mori and Sakano, 2021). During the primary projection of OSNs, odor information is sorted into two separate qualities, aversive and attractive, along the D/V axis of the OB using repulsive axon guidance molecules Nrp2 and Sema3F. Nrp2^–^/Sema3F^+^ axons target to the dorsal OB, while Nrp2^+^/Sema3F^–^ axons target to the ventral OB (Takeuchi et al., 2010). It has been reported that aversive information collected in the dorsal OB is transmitted to the pmCoA (Miyamichi et al., 2011; Root et al., 2014), whereas attractive social information is transmitted from the postero-ventral OB to the anterior medial-amygdala (aMeA) (Inokuchi et al., 2017). For this segregation of secondary projection, the same set of guidance molecules Nrp2/Sema3F as for the primary projection, are involved in MC migration and targeting to the amygdala. The Nrp2^+^ MCs are guided to the ventral OB by repulsive interactions with Sema3F and send their axons to the aMeA. In contrast, the Nrp2^–^ MCs remain in the dorsal OB and target to the pmCoA (Inokuchi et al., 2017).

In the MC-specific conditional KO of Nrp2, attractive social behaviors, e.g., ultrasonic vocalization of male mice toward female scents, are perturbed (Inokuchi et al., 2017). Interestingly, innate fear/aversive responses are not affected, indicating that a separate axon guidance system is responsible for directing the Nrp2^–^ MC axons to the pmCoA. Gain-of-function experiments with electroporation of the *Nrp2* gene demonstrated that activation of the *Nrp2* alone in the embryonic MCs is sufficient to induce the segregation of aversive and attractive pathways. Forced expression of the *Nrp2* in the Nrp2^–^ MCs cause them to migrate to the ventral OB and send their axons to the aMeA, but not to the pmCoA.

How are the OSN axons and MC dendrites properly connected within the glomeruli? To ensure finding the correct partner for functional synapse formation, there are two different hypotheses. One is the specificity model that assumes specific matching based on the OR identity. The other is the proximity model which assumes that the OSN axons synapse with nearest-neighboring MC dendrites based on the physical distances. Using various mutant mice in which the glomerular map formation is perturbed, Nishizumi et al. (2019) found that MC dendrites choose their partner-glomeruli based on the proximity without recognizing the OR identity. Thus, co-regulation of OSN projection and MC migration using the same guidance system, Nrp2/Sema3F, is key to proper pairing of the primary-neuron axons and secondary-neuron dendrites.

## Activity-Dependent Modulation of Odor Qualities

Odor qualities are innately determined, but can be changed by imprinted memory during the neonatal critical period (Inoue et al., 2021). Ducklings follow the first moving object after hatching, recognizing it as a parental bird (Lorenz, 1935). Eye mask experiments performed during the critical period, which allow activity-dependent circuit formation to occur, are widely known (Wiesel and Hubel, 1963). Although these experiments were reported decades ago, not so much is known about plastic changes in the sensory system at the molecular level.

Recently, Inoue et al. (2018) identified the signaling molecules Sema7A/PlxnC1 for olfactory imprinting. To precisely define the olfactory critical period, unilateral naris-occlusion experiments were performed: occlusion was started right after birth and continued to various time points. When the occluded naris was reopened before postnatal day 7 (P7), no obvious defects were observed for odor sensing and perception. However, if the occlusion was continued after P8, synapse formation and dendrite selection were significantly delayed. Furthermore, social behaviors were perturbed later in life: the mice avoided stressful interactions with unfamiliar mice. In order to identify the responsible molecules for imprinting, Inoue et al. (2018) examined various signaling molecules for their expression in the embryonic OE and OB. As a result, a pair of molecules, Sema7A and its receptor PlxnC1, were found to be good candidates.

Sema7A is expressed in the axon termini of OSNs in an activity-dependent manner and PlxnC1 is localized to the MC dendrites only during the first week after birth. KO experiments demonstrated that unlike other Sema-family proteins, blockage of Sema7A signaling did not affect olfactory map formation but affected post-synaptic events and dendrite selection in MCs. In the gain-of-function experiment, exposure to a particular odorant during the critical period resulted in enlargement of the responding glomeruli and increased sensitivity to the conditioned odor. In addition, the mice demonstrated lasting interest to the imprinted odor in the habituation/dishabituation test. In the stress-induced hyperthermia test, imprinting was found to have a stress-easing effect, reducing the plasma concentrations of the stress hormone ACTH. It should be mentioned that these effects can be found even for aversive odorants, e.g., 4-methyl thiazole (a derivative of TMT). Then, what is responsible for imposing the positive quality on the imprinted odor memory?

Among various hormones expressed in neonates, Inoue et al. (2021) focused on oxytocin, because it is needed for smooth social interactions (Bosch and Young, 2018). When the oxytocin KO was conditioned to 4MT, the sensitivity to 4MT was increased, but the stress-reducing effect was not observed (Inoue et al., 2021). The KO mice did not become accustomed to the stranger mice in the social-memory test. Interestingly, when oxytocin was administrated in neonates by intraperitoneal injection, impaired social responses were improved although this rescue effect was not seen when the injection was given after the critical period. These experiments indicate that the oxytocin in neonates is needed for imposing the positive quality on imprinted memory. It will be interesting to examine whether the aversive quality can be imprinted to the conditioned odor if the stress hormone is administrated during the critical period.

## Two Processing Streams of Olfactory Information

Each OB contains two mirror-symmetrical maps of glomeruli: lateral map in the dorsolateral region and medial map in the ventromedial region (Figure 1A; Nagao et al., 2000; Mori and Sakano, 2011). We recently proposed a hypothesis that the medial map of the OB receives top-down attention signals during eating behavior to enhance the processing of retronasal/interoceptive odor inputs from foods in the oral cavity. In contrast, the lateral map of the OB receives the attention signals during object-searching behavior to enhance the processing of orthonasal/exteroceptive odor inputs from the environment (Mori and Sakano, 2022).

We hypothesize that the medial map of the OB conveys exhalation-phased retronasal odor information mainly to the medial areas of the OC including the medial and posterior parts of the AON (AONm, AONp), ventral and dorsal tenia tecta (TTv, TTd), and dorsal peduncular cortex (DP) (magenta arrows in Figures 1A,B; Haberly and Price, 1978; Brunjes et al., 2005; Hirata et al., 2019). We call these pathways “medial retronasal/interoceptive stream” and assume that the medial areas of the OC mainly process the retronasal/interoceptive odor information for inducing autonomic and pituitary-hormone responses to food in the oral cavity or digestive tract.

Figure 1 also shows examples of subcortical and extra-telencephalic targets of pyramidal cells in the medial OC areas. For example, the rostromedial part of the olfactory tubercle (OTrm) receives a direct input from the medial map of the OB and indirect olfactory inputs *via* TTv, suggesting that the OTrm processes retronasal odor information. The OTrm sends inhibitory outputs to the rostromedial part of the ventral pallidum (VPrm) (Heimer et al., 1987; Ikemoto, 2007) and di-synaptically disinhibit neurons in the medial part of the mediodorsal nucleus of thalamus (MDm), posteromedial part of the ventral tegmental area (VTApm), and lateral hypothalamus (LH) (Mengual et al., 2016). These pathways indicate that the OTrm is involved in transforming retronasal odor inputs into aversive or attractive behavioral responses (Ikemoto, 2007; Murata et al., 2015; Wright and Wesson, 2021).

The DP and TTd are located in the medial part of the olfactory peduncle. They project to the dorsomedial hypothalamus (DMH) that targets to sympathetic premotor neurons in the brainstem (Figure 1; Nakamura and Morrison, 2022). This observation suggests that the DP and TTd transform the retronasal odor information to sympathetic stress-responses.

We hypothesize that the lateral map mainly conveys inhalation-phased orthonasal/exteroceptive odor information to the lateral part of the OC including the external and lateral parts of the AON (AONe, AONl), ventral and dorsal parts of the anterior piriform cortex (APCv, APCd), posterior piriform cortex (PPC), cortical amygdaloid areas (CoA), and lateral entorhinal area (ENTl) (blue arrows in Figure 1; Igarashi et al., 2012; Mori et al., 2013). We name these pathways “lateral orthonasal/exteroceptive stream” and presume that the lateral areas of the OC mainly process orthonasal/exteroceptive odor information for inducing either aversive or attractive behavioral responses.

The lateral map of the OB also projects directly to subcortical targets such as the aMeA and lateral part of the olfactory tubercle (OTl), suggesting that these areas process orthonasal odor information. The OTl projects their inhibitory axons to the rostrolateral part of the ventral pallidum (VPrl) that sends inhibitory axons to the central part of the mediodorsal nucleus of the thalamus (MDc), anterolateral part of the ventral tegmental area (VTAal) and LH (Ikemoto, 2007; Mengual et al., 2016). These pathways indicate that OTl is involved in transforming the orthonasal odor inputs to motivated behavioral responses. Figure 1 also shows that lateral areas of the OC project to the basolateral amygdaloid complex (BLA) and central amygdaloid nuclei (CeAl, CeAm) to induce emotional behavioral responses to the orthonasal odor input.

## External and Internal Odors Inducing Stress Responses

Stress-inducing sensory information can be classified into two categories: internal stressors such as low body temperature and external ones such as the sight of a predator (Ulrich-Lai and Herman, 2009). The olfactory system is characteristic in that it processes both categories of odors. External odors that induce stress responses include orthonasal odors of predators (Kobayakawa et al., 2007; Kondoh et al., 2016), competitors searching for food or mating partners, harmful substances, and places where an aversive situation is present. Internal stress-odors include the retronasal odors of spoiled food and inedible things in the oral cavity. Our model shown in Figure 1 suggests that the lateral orthonasal/exteroceptive stream and the medial retronasal/interoceptive stream mainly process the external and internal odor-stress information, respectively.

**FIGURE 2:**
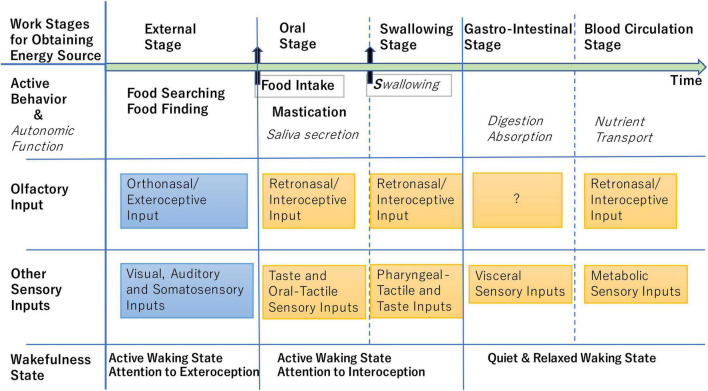
Schematic diagram illustrating the stage-by-stage workflow for obtaining energy source. Active behavior and autonomic function in each stage are listed in the second row, while possible external and internal sensory inputs including stressors that occur in each stage are shown in the third and fourth rows. At the external stage, the brain shows active waking state (fifth row) and attends to the orthonasal/exteroceptive odor information and other exteroceptive sensory information (blue boxes). At the oral and swallowing stages, the brain shows active waking state attending to the retronasal/interoceptive odor information and other interoceptive sensory information (orange boxes). At the gastrointestinal and blood-circulation stages, the brain shows quiet and relaxed state with reduced attention to the exteroceptive and interoceptive sensory inputs (Brown et al., 2012). At any stage, retronasal detection of aversive odors indicating metabolic abnormality may alert the animal and induce the stress responses (Bollen and Beikler, 2012; Poniewierka et al., 2022).

As illustrated in Figure 2, eating behavior consists of multiple stages: food searching (external stage) precedes food ingestion (oral and swallowing stages), and ingestion is followed by digestion of food and absorption of nutrients (gastrointestinal stage) (Shepherd, 2012). The external stage is tightly coupled with external odor inputs whereas the subsequent stages are coupled with internal odor inputs (Shepherd, 2012; Kikuta et al., 2017; Mori and Sakano, 2022). Any factor that interrupts the stage-by-stage workflow causes a serious threat to energy homeostasis. Therefore, the brain perceives odor inputs that predict the interruption of the workflow as a stress odor.

During the external stage, detection of predator odors causes the animal to cease food-searching, while spoiled food odor in the environment alerts the animal to not ingest the food. We hypothesize that MCs and TCs in the aversive D_I_ domain and the fear D_II_ domain in the lateral map of the OB convey the external odor signals either directly or indirectly *via* AON and APC to the negative valence networks in the AmPir and pmCoA. The AmPir neurons responding to predator odors activate the corticotropin-releasing hormone (CRH) neurons in the paraventricular nucleus of the hypothalamus (PVH) for stress-hormone release (Kondoh et al., 2016). The odor signals in the AmPir and pmCoA are further transmitted to the BLA and CeA to induce aversive behavioral responses (Figure 1). Since amygdaloid areas send axons to the medial prefrontal cortex including the DP and TTd, amygdala-DP/TTd-DMH is a candidate pathway for the sympathetic stress responses to external stress odors.

During the oral swallowing and gastrointestinal stages, internal odor inputs play a key role in inducing behavioral and autonomic responses. During the oral stage, retronasal odors from spoiled food in the oral cavity generate alert signals that activate stress responses and tell the animal to stop chewing and to spit the food out. Then, which neural pathway is responsible for the stress responses to retronasal odors? It has been shown that the DP and TTd in the medial OC play a role as a hub in transforming a variety of psychological stress-signals to sympathetic thermogenesis and cardiovascular responses *via* the pathway through DMH, rostral medullary raphe region (rMR), and rostral ventrolateral medulla (RVLM) (Figure 1; Kataoka et al., 2020; Nakamura and Morrison, 2022). Because the DP and TTd receive rich inputs from MCs and TCs in the medial map of the OB, we speculate that stress-inducing internal odors activate the DP and TTd and transmit aversive odor signals to the DMH-rMR/RVLM-sympathetic pathway, thus inducing the sympathetic responses.

The afferent and efferent connections of the DP and TTd (Figure 1) suggest that they integrate the retronasal odor signals from the medial map of the OB with other psychological stress signals to decide whether the food in the mouth should be swallowed or spit out. The OB-DP/TTd-DMH pathway appears to be the shortest neural pathway for internal odor information to induce the sympathetic stress responses.

In the behavior of eating natural foods such as nuts, rodents rapidly repeat the sequence of “orthonasal examination of the external food (external stage in Figure 2),” “biting and putting a piece of food into the mouth,” and “chewing the food and detecting the retronasal odor of the food (oral stage)” to eat edible parts and spit out inedible parts. We hypothesize that at the external stage, the brain attends to the lateral orthonasal/exteroceptive stream to make a decision whether or not to take the food into the mouth. At the external stage, orthonasal odors of spoiled food may be processed mainly by the lateral stream and activate negative valence circuits in the amygdala, which then transmits the negative valence signal to the DP/TTd-DMH pathway to induce sympathetic stress responses and to decide not to put the food into the mouth. We also assume that, at the subsequent oral stage, the brain rapidly switches its attention to the retronasal/interoceptive stream to decide whether the piece of food should be spitted out or not. At the oral stage, retronasal odor signals of spoiled food may be processed mainly by the medial map of the OB and directly transmitted to the DP/TTd to activate the sympathetic stress pathway and to induce the spitting behavior. How does the brain switch over its attention from the orthonasal stream to the retronasal stream in accord with the current behavior? This is an important question to be elucidated in the future.

## Olfactory Processing Under Homeostatic Challenges

Physiological conditions of the body such as energy metabolism, salt and water balance, and body temperature are continuously monitored by interoceptive sensory systems in the hypothalamus, brainstem, and spinal cord to maintain the homeostasis of the body. Under homeostatic challenges, it is of critical importance for the animal to adaptively change sensory processing, behavioral outputs, and neuroendocrine/autonomic responses (Ulrich-Lai and Herman, 2009). Interoceptive signals of homeostatic challenges are sent to the cortical and subcortical areas to adaptively modulate the processing of sensory information and generation of behavioral outputs.

Hunger and thirst are the internal physiological stress and homeostatic challenges essential for driving food/water-searching and ingestion behaviors (Augustine et al., 2020). Recent studies demonstrate that hunger is constantly monitored by AgRP neurons in the arcuate nucleus of the hypothalamus and that their output is transmitted to a wide range of areas in the forebrain including olfactory cortical areas (Betley et al., 2015). Continuous recording of AgRP neurons indicates that long-lasting suppression of their activity occurs in phase with satiation from the food (Zimmerman and Knight, 2020).

Interestingly, the activity of AgRP neurons transiently decreases during the pre-eating food-searching stage when the animal detects sensory cues of food in the environment including the orthonasal odor cue (Chen et al., 2015). Also, at stages when food is in the oral cavity or pharynx (Figure 2), the hunger activity of AgRP neurons is transiently modulated by sensory inputs of gustatory, oropharyngeal somatosensory, and retronasal olfactory signals from the food in the oropharyngeal region (Chen et al., 2015). At the stage when food is in the stomach and intestine, vagal afferent signals and neuroendocrine signals from the gastrointestinal tract elicit long-lasting suppression of the hunger activity of AgRP neurons as demonstrated by intragastric injection of nutrients (Beutler et al., 2017). The stage-specific modulation of AgRP-neuron activity indicates that brainwide cortical and subcortical sensory networks coordinately transmit sensory signals from the food to the AgRP neurons in the hypothalamus.

During the pre-eating stage, orthonasal/exteroceptive odor signals that predict the presence of food may be detected by the lateral OB map and conveyed *via* the lateral stream to AgRP neurons. During the oral stage, retronasal/interoceptive odor signals that predict the nutrition may be detected by the medial OB map and conveyed *via* the medial stream to AgRP neurons. These hypotheses are yet to be verified by future experiments.

Optogenetic activation of hunger-sensitive AgRP neurons indicates that the activity of AgRP neurons is aversive to mice and that these neurons provide brainwide networks with the negative-valence signals to induce motivated behavior toward external or internal sensory cues associated with food (Betley et al., 2015). These neurons play a critical role in controlling active behaviors during both the external stage (foraging) and internal oral stage (chewing and swallowing) of the eating-associated behavioral sequence (Figure 2; Chen et al., 2015). It has been shown that olfactory sensory processing is influenced by metabolic signals such as ghrelin and insulin that are changed by the behavioral sequence (Palouzier-Paulignan et al., 2012). However, it is yet to be clarified which neural circuit transmits the AgRP neuron activity to the OC to modulate the processing of food odors.

Activity of the thirst-sensing preoptic-neurons in the hypothalamus are also aversive (Betley et al., 2015; Allen et al., 2017, 2019; Leib et al., 2017) and plays a critical role in driving thirst-motivated behaviors. In water-restricted mice, water-predicting exteroceptive odor-cues elicit specific activity first in the olfactory-system neurons (e.g., AON and APC) and then in the neurons across brain-wide networks. After satiation with water, this neural activity disappears. However, optogenetic activation of the hypothalamic thirst-sensing neurons retuned the brainwide activity to the level of the pre-satiation state (Allen et al., 2017). This indicates that the thirst-sensing hypothalamic activity reaches the olfactory circuitry and regulates the way in which the signals of water-predicting odor cue are processed and transformed into appropriate behavioral outputs. Future studies will elucidate the circuit mechanism of how the thirst-sensing signals regulate olfactory sensory processing and behavioral decisions.

## Discussion

In the mammalian olfactory system, odor information can be sorted into two distinct qualities, positive (pleasant/attractive) and negative (stressful/aversive) during the primary projection of OSNs to the OB. For instinctive decisions, odor signals are directly transmitted to the valence regions in the amygdala *via* hard-wired circuits. Such decisions are biologically correct as they are the consequence of natural selection during evolution. Basic olfactory decisions for the survival of individuals and species are innately determined and genetically programmed. The mammalian brain also evaluates input sensory signals based on the previous scene of olfactory memory. In either case, whether innate or learned, when the quality of input information is positive, the animal proceeds with the goal-directed behavior, and becomes satisfied and relaxed if it attains the goal. In contrast, when the input information is negative, the stress response occurs occasionally with increased release of stress-hormones and the new goal-oriented strategy is built to improve the situation. The stress is an alert signal, not only to avoid the harmful object or to improve the situation but also to motivate the animal to accomplish the goal, when its current situation is not satisfactory. Neuro-transmitters and brain hormones are expressed for taking an action to remove the danger so that the host can return to an improved state. The brain perceives the sensory signals as stress, which may be harbingers of future danger. The stress signals drive motivated behaviors for survival. Thus, the stress response is quite important to keep the physiological homeostasis of the body and to protect the animal from various crises. It will be interesting to study how the brain measures the distance from the present state to the goal state and how it builds a strategy to achieve this purpose. Although we generally have a negative impression of stress, it is a motivational message from the sensory systems to improve a situation. If the animal ignores stress signals, they will simply become sick, die in the danger, or remain in the current poor situation.

In addition to negative signals inducing stress, positive valence-signals also inspire the animal to achieve goals. The goal-oriented attractive behavior is induced by a desire mechanism that is mediated by dopamine, adrenaline, and other hormones. Thus, the positive-valence signals are not just for satisfaction, but also to induce the attractive behavior to accomplish a goal. Recent studies demonstrated that during odor-reward association learning, OT neurons rapidly acquire reward-selective responses and encode the positive-valence of conditioned odor (Gadziola et al., 2015; Millman and Murthy, 2020; Midroit et al., 2021) and that dopaminergic projection from the VTA to the medial part of the OT mediates naturalistic reward processes (Zhang et al., 2017). Future studies may elucidate the functional difference between the medial and lateral OB-AON/TT-OT-VP-VTA pathways (Figure 1) in the odor-reward association learning with regard to retronasal and orthonasal odor inputs.

Olfactory circuitry in the OB, OC, CoA, and OT plays a crucial role in perceiving the external and internal odors and judging the valence of odor inputs. When the animal inhales the orthonasal odors of a predator, the lateral stream may process the external odor information for predicting the presence of danger and driving aversive behaviors to avoid it. When the animal perceives retronasal odors of tasty foods in the oral cavity, the medial stream may process the internal odor information for predicting nutrients and driving eating behaviors, e.g., chewing and swallowing, as well as secreting saliva and gastric fluid. Although the immediate reflex-like responses to internal stressors are generated unconsciously by the autonomic networks in the hypothalamus, brainstem, and spinal cord (Ulrich-Lai and Herman, 2009; Sklerow et al., 2019), cortical and subcortical mechanisms of perceiving the exteroceptive sensory inputs and their evaluation operate only in awake conscious states. Future studies will elucidate the detailed mechanisms of how the olfactory network in the OB and OC controls the autonomic networks in the hypothalamus, brainstem, and spinal cord, and how the autonomic afferent signals influence sensory processing in the OB and OC. The mouse olfactory system provides an excellent model for our understanding of how the transition between the hunger and satiety states, or between the thirst and quenched states, influences processing of sensory information and generation of behavioral decisions.

## Author Contributions

Both authors listed have made a substantial, direct, and intellectual contribution to the work, and approved it for publication.

## Conflict of Interest

The authors declare that the research was conducted in the absence of any commercial or financial relationships that could be construed as a potential conflict of interest.

## Publisher’s Note

All claims expressed in this article are solely those of the authors and do not necessarily represent those of their affiliated organizations, or those of the publisher, the editors and the reviewers. Any product that may be evaluated in this article, or claim that may be made by its manufacturer, is not guaranteed or endorsed by the publisher.

## References

[B1] Allen W. E. Chen M. Z. Pichamoorthy N. Tien R. H. Pachitariu M. Luo L. (2019). Thirst regulates motivated behavior through modulation of brainwide neural population dynamics. *Science* 364:253. 10.1126/science.aav3932 30948440 PMC6711472

[B2] Allen W. E. DeNardo L. A. Chen M. Z. Liu C. D. Loh K. M. Fenno L. E. (2017). Thirst-associated preoptic neurons encode an aversive motivation drive. *Science* 357 1149–1155. 10.1126/science.aan6747 28912243 PMC5723384

[B3] Allen Institute for Brain Science (2004). *Allen Mouse Brain Atlas, Reference Atlas.* Washington, DC: Allen Institute for Brain Science.

[B4] Augustine V. Lee S. Oka Y. (2020). Neural control and modulation of thirst, sodium appetite, and hunger. *Cell* 180 25–32. 10.1016/j.cell.2019.11.040 31923398 PMC7406138

[B5] Betley J. N. Xu S. Cao Z. F. H. Gong R. Magnus C. J. Yu Y. (2015). Neurons for hunger and thirst transmit a negative-valence teaching signal. *Nature* 521 180–185. 10.1038/nature14416 25915020 PMC4567040

[B6] Beutler L. R. Chen Y. Ahn J. S. Lin Y.-C. Essner R. A. Knight Z. A. (2017). Dynamics of gut-brain communication underlying hunger. *Neuron* 96 461–475. 10.1016/j.neuron.2017.09.043 29024666 PMC5691364

[B7] Bollen C. M. L. Beikler T. (2012). Halitosis: the multidisciplinary approach. *Int. J. Oral Sci.* 4 55–63. 10.1038/ijos.2012.39 22722640 PMC3412664

[B8] Bosch O. J. Young L. J. (2018). Oxytocin and social relationships: from attachment to bond disruption. *Curr. Top. Behav. Neurosci.* 35 97–117. 10.1007/7854_2017_10 28812266 PMC5815947

[B9] Brown R. E. Basheer R. McKenna J. T. Strecker R. E. McCarley R. W. (2012). Control of sleep and wakefulness. *Physiol. Rev*. 92 1087–1187. 22811426 10.1152/physrev.00032.2011 PMC3621793

[B10] Brunjes P. C. Illig K. R. Meyer E. A. (2005). A field guide to the anterior olfactory nucleus/cortex. *Brain Res. Rev.* 50 305–335. 10.1016/j.brainresrev.2005.08.005 16229895

[B11] Buck L. Axel R. (1991). A novel multigene family may encode odorant receptors: a molecular basis for odor recognition. *Cell* 65 175–187. 10.1016/0092-8674(91)90418-x 1840504

[B12] Chen Y. Lin Y.-C. Kuo T.-W. Knight Z. A. (2015). Sensory detection of food rapidly modulates arcuate feeding circuits. *Cell* 160 829–841. 10.1016/j.cell.2015.01.033 25703096 PMC4373539

[B13] Gadziola M. A. Tylicki K. A. Christian D. L. Wesson D. W. (2015). The olfactory tubercle encodes odor valence in behaving mice. *J. Neurosci.* 35 4515–4527. 10.1523/JNEUROSCI.4750-14.2015 25788670 PMC6605138

[B14] Haberly L. B. Price J. L. (1978). Association and commissural fiber systems of the olfactory cortex of the rat: II. Systems originating in the olfactory peduncle. *J. Comp. Neurol.* 178 781–808. 10.1002/cne.901810407 690285

[B15] Heimer L. Zaborszky L. Zahn D. S. Alheid G. F. (1987). The ventral striatopallidothalamic projection: I. The striatopallidal link originating in the striatal parts of the olfactory tubercle. *J. Comp. Neurol*. 255 571–591. 10.1002/cne.902550409 3029188

[B16] Herman J. P. McKlveen J. M. Ghasal S. Kopp B. Wulsin A. Makinson R. (2016). Regulation of the hypothalamic-pituitary-adrenocortical stress response. *Compr. Physiol.* 6 603–621. 10.1002/cphy.c150015 27065163 PMC4867107

[B17] Hirata T. Shioi G. Abe T. Kiyonari H. Kato S. Kobayashi K. (2019). A novel birthdate labeling method reveals segregated parallel projections of mitral and external tufted cells in the main olfactory system. *eNeuro* 6:ENEURO.0234-19.2019. 10.1523/ENEURO.0234-19.2019 PMC6868177 31672846

[B18] Igarashi K. M. Ieki N. An M. Yamaguchi Y. Nagayama S. Kobayakawa K. (2012). Parallel mitral and tufted cell pathways route distinct odor information to different targets in the olfactory cortex. *J. Neurosci.* 32 7970–7985. 10.1523/JNEUROSCI.0154-12.2012 22674272 PMC3636718

[B19] Ikemoto S. (2007). Dopamine reward circuitry: two projection systems from the ventral midbrain to the nucleus accumbens-olfactory tubercle complex. *Brain Res. Rev*. 56 27–78. 17574681 10.1016/j.brainresrev.2007.05.004 PMC2134972

[B20] Imai T. Suzuki M. Sakano H. (2006). Odorant receptor-derived cAMP signals direct axonal targeting. *Science* 314 657–661. 10.1126/science.1131794 16990513

[B21] Inokuchi K. Imamura F. Takeuchi H. Kim R. Okuno H. Nishizumi H. (2017). Nrp2 is sufficient to instruct circuit formation of mitral-cells to mediate odor-induced attractive social responses. *Nat. Commun*. 8:15977. 10.1038/ncomms15977 28731029 PMC5525001

[B22] Inoue N. Nishizumi H. Naritsuka H. Kiyonari H. Sakano H. (2018). Sema7A/PlxnC1 signaling triggers the activity-dependent olfactory synapse formation. *Nat. Commun.* 9:1842. 10.1038/s41467-018-04239-z 29743476 PMC5943276

[B23] Inoue N. Nishizumi H. Ooyama R. Mogi K. Nishimori K. Kikusui T. (2021). The olfactory critical period is determined by activity-dependent Sema7A/PlxnC1 signaling within glomeruli. *eLife* 10:e65078. 10.7554/eLife.65078 33780330 PMC8007213

[B24] Kataoka N. Shima Y. Nakajima K. Nakamura K. (2020). A central master driver of psychosocial stress responses in the rat. *Science* 367 1105–1112. 10.1126/science.aaz4639 32139538

[B25] Kikuta S. Matsumoto Y. Kuboki A. Nakayama T. Asaka D. Otori N. (2017). Longer latency of sensory response to intravenous odor injection predicts olfactory neural disorder. *Sci. Rep.* 6:35361. 10.1038/srep35361 27734933 PMC5062120

[B26] Kobayakawa K. Kobayakawa R. Matsumoto H. Oka Y. Imai T. Ikawa M. (2007). Innate versus learned odour processing in the mouse olfactory bulb. *Nature* 450 503–508. 10.1038/nature06281 17989651

[B27] Kondoh K. Lu Z. Ye X. Olson D. P. Lowell D. D. Buck L. B. (2016). A specific area of olfactory cortex involved in stress hormone responses to predator odors. *Nature* 532 103–106. 10.1038/nature17156 27001694 PMC5094457

[B28] Leib D. E. Zimmerman C. A. Poormoghaddam A. Huey E. L. Ahn J. S. Lin Y.-C. (2017). The forebrain thirst circuit drives drinking through negative reinforcement. *Neuron* 96 1272–1281. 10.1016/j.neuron.2017.11.041 29268095 PMC5940335

[B29] Lorenz K. (1935). Der Kumpan in der Umwelt des Vogels. Der Artgenosse als auslösendes Moment sozialer Verhaltensweisen. *J. Ornithol.* 83 289–413.

[B30] Mengual E. Prensa L. Tripathi A. Cebrian C. Mongia S. (2016). “Comparative analysis of he axonal collateralization patterns of basal ganglia output nuclei in the rat,” in *Axons and Brain Architecture*, ed. Rockland K. S. (London: Academic Press), 47–68.

[B31] Midroit M. Chalençon L. Renier N. Milton A. Thevenet M. Sacquet J. (2021). Neural processing of the reward value of pleasant odorants. *Curr. Biol*. 31 1592–1605.e9. 10.1016/j.cub.2021.01.066 33607032 PMC9291255

[B32] Millman D. J. Murthy V. N. (2020). Rapid learning of odor-value association in olfactory striatum. *J. Neurosci.* 40 4335–4347. 10.1523/JNEUROSCI.2604-19.2020 32321744 PMC7252476

[B33] Miyamichi K. Amat F. Moussavi F. Wang C. Wickersham I. Wall N. R. (2011). Cortical representations of olfactory input by trans-synaptic tracing. *Nature* 472 191–196. 10.1038/nature09714 21179085 PMC3073090

[B34] Mombaerts P. Wang F. Dulac C. Chao S. K. Nemes A. Mendelsohn M. (1996). Visualizing an olfactory sensory map. *Cell* 87 675–686. 10.1016/s0092-8674(00)81387-2 8929536

[B35] Mori K. Manabe H. (2014). “Unique characteristics of the olfactory system,” in *The Olfactory System*, ed. Mori K. (Tokyo: Springer), 1–18.

[B36] Mori K. Manabe H. Narikiyo K. Onisawa N. (2013). Olfactory consciousness and gamma oscillation coupling across the olfactory bulb, olfactory cortex, and orbitofrontal cortex. *Front. Psychol.* 4:743. 10.3389/fpsyg.2013.00743 24137148 PMC3797617

[B37] Mori K. Sakano H. (2011). How is the olfactory map formed and interpreted in the mammalian brain? *Annu. Rev. Neurosci*. 34 467–499. 10.1146/annurev-neuro-112210-112917 21469960

[B38] Mori K. Sakano H. (2021). Olfactory circuitry and behavioral decisions. *Annu. Rev. Physiol.* 83 231–256. 10.1146/annurev-physiol-031820-092824 33228453

[B39] Mori K. Sakano H. (2022). Processing of odor information during the respiratory cycle in mice. *Front. Neural Circuits* 16:861800. 10.3389/fncir.2022.861800 35431818 PMC9008203

[B40] Murata K. Kanno M. Ieki N. Mori K. Yamaguchi M. (2015). Mapping of learned odor-induced motivated behaviors in the mouse olfactory tubercle. *J. Neurosci.* 35 10581–10599. 10.1523/JNEUROSCI.0073-15.2015 26203152 PMC6605114

[B41] Nagao H. Yoshihara Y. Mitsui S. Fujisawa H. Mori K. (2000). Two mirror- image sensory maps with domain organization in the mouse main olfactory bulb. *Neuroreport* 11 3023–3027. 10.1097/00001756-200009110-00039 11006987

[B42] Nakamura K. Morrison S. F. (2022). Central sympathetic network for thermoregulatory responses to psychological stress. *Auton. Neurosci.* 237:102918. 34823147 10.1016/j.autneu.2021.102918

[B43] Nakashima A. Takeuchi H. Imai T. Saito H. Kiyonari H. Abe T. (2013). Agonist-independent GPCR activity regulates anterior-posterior axon targeting of olfactory sensory neurons. *Cell* 154 1314–1325. 10.1016/j.cell.2013.08.033 24034253 PMC7394037

[B44] Nishizumi H. Miyashita A. Inoue N. Inokuchi K. Aoki M. Sakano H. (2019). Primary dendrites of mitral cells synapse unto neighboring glomeruli independent of their odorant receptor identity. *Commun. Biol.* 2:14. 10.1038/s42003-018-0252-y 30652126 PMC6325062

[B45] Nishizumi H. Sakano H. (2019). Agonist-independent GPCR activity and receptor-instructed axonal projection in the mouse olfactory system. *Future Med. Chem.* 11 3091–3096. 10.4155/fmc-2019-0178 31838900

[B46] Palouzier-Paulignan B. Lacroix M.-C. Aimé P. Baly C. Caillol M. Congar P. (2012). Olfaction under metabolic influences. *Chem. Senses* 37 767–797. 10.1093/chemse/bjs059 22832483 PMC3529618

[B47] Poniewierka E. Pleskacz M. Łuc-Pleskacz N. Kłaniecka-Broniek J. (2022). Halitosis as a symptom of gastroenterological diseases. *Gastroenterol. Rev.* 17 17–21. 10.5114/pg.2022.114593 35371354 PMC8942002

[B48] Rasmussen S. G. Choi H. J. Fung J. J. Pardon E. Casarosa P. Chae P. S. (2011). Structure of a nanobody-stabilized active state of the β2 adrenoceptor. *Nature* 469 175–180. 21228869 10.1038/nature09648 PMC3058308

[B49] Root C. M. Denny C. A. Hen R. Axel R. (2014). The participation of cortical amygdala in innate, odour-driven behaviour. *Nature* 515 269–273. 10.1038/nature13897 25383519 PMC4231015

[B50] Saito H. Nishizumi H. Suzuki S. Matsumoto H. Ieki N. Abe T. (2017). Immobility responses are induced by photoactivation of a single glomerular species responsive to fox odor TMT. *Nat. Commun*. 8:16011. 10.1038/ncomms16011 28685774 PMC5504302

[B51] Sakano H. (2010). Neural map formation in the mouse olfactory system. *Neuron* 67 530–542. 20797531 10.1016/j.neuron.2010.07.003

[B52] Serizawa S. Miyamichi K. Nakatani H. Suzuki M. Saito M. Yoshihara Y. (2003). Negative feedback regulation ensures the one receptor-one olfactory neuron rule in mouse. *Science* 302 2088–2094. 10.1126/science.1089122 14593185

[B53] Serizawa S. Miyamichi K. Takeuchi H. Yamagishi Y. Suzuki M. Sakano H. (2006). A neuronal identity code for the odorant receptor-specific and activity-dependent axon sorting. *Cell* 127 1057–1069. 10.1016/j.cell.2006.10.031 17129788

[B54] Shepherd G. M. (2012). *Neurogastronomy. How the Brain Creates Flavor and Why it Matters.* New York, NY: Columbia Univ. Press.

[B55] Sklerow M. Dayan E. Browner N. (2019). Functional neuroimaging of the central autonomic network: recent developments and clinical implications. *Clin. Auton. Res.* 29 555–566. 10.1007/s10286-018-0577-0 30470943 PMC6858471

[B56] Takeuchi H. Inokuchi K. Aoki M. Suto F. Tsuboi A. Matsuda I. (2010). Sequential arrival and graded secretion of Sema3F by olfactory neuron axons specify map topography at the bulb. *Cell* 141 1056–1067. 10.1016/j.cell.2010.04.041 20550939

[B57] Ulrich-Lai Y. M. Herman J. P. (2009). Neural regulation of endocrine and autonomic stress responses. *Nat. Rev. Neurosci.* 10 397–409. 10.1038/nrn2647 19469025 PMC4240627

[B58] Wiesel T. N. Hubel D. H. (1963). Single-cell responses in striate cortex of kittens deprived of vision in one eye. *J. Neurophysiol.* 26 1003–1017. 14084161 10.1152/jn.1963.26.6.1003

[B59] Wright K. N. Wesson D. W. (2021). The tubular striatum and the nucleus accumbens distinctly represent reward-taking and reward-seeking. *J. Neurophysiol*. 125 166–183. 10.1152/jn.00495.2020 33174477 PMC8087377

[B60] Zhang Z. Liu Q. Wen P. Zhang J. Rao X. Zhou Z. (2017). Activation of the dopaminergic pathway from VTA to the medial olfactory tubercle generates odor preference and reward. *eLife* 6:e25423. 10.7554/eLife.25423 29251597 PMC5777817

[B61] Zimmerman C. A. Knight Z. A. (2020). Layers of signals that regulate appetite. *Curr. Opin. Neurobiol.* 64 79–88. 10.1016/j.conb.2020.03.007 32311645 PMC7572475

